# Herbal Spices as Food and Medicine: Microscopic Authentication of Commercial Herbal Spices

**DOI:** 10.3390/plants13081067

**Published:** 2024-04-10

**Authors:** Amjad Khan, Mushtaq Ahmad, Amir Sultan, Raees Khan, Jamil Raza, Sheikh Zain Ul Abidin, Siraj Khan, Muhammad Zafar, Mohammad N. Uddin, Mohsin Kazi

**Affiliations:** 1 Department of Plant Sciences, Quaid-i-Azam University, Islamabad 45320, Pakistan; amjadkhan@bs.qau.edu.pk (A.K.); jraza@bs.qau.edu.pk (J.R.);; 2 National Herbarium of Pakistan (Stewart Collection), Plant Genetic Resources Institute, National Agricultural Research Centre, PARC, Islamabad 30341, Pakistan; amirsultan_2000@yahoo.com; 3 Institute of Biological Sciences, Gomal University D I Khan, Dera Ismail Khan 29050, Pakistan; zain@bs.qau.edu.pk; 4 Qarshi Herb Research Center at Qarshi Industries (Pvt.) Ltd., Lahore 22610, Pakistan; khansiraj60@yahoo.com; 5 Department of Eastern Medicine, Qarshi University, Lahore 54000, Pakistan; 6 College of Pharmacy, Mercer University, 3001 Mercer University Drive, Atlanta, GA 30341, USA; uddin_mn@mercer.edu; 7 Department of Pharmaceutics, College of Pharmacy, King Saud University, P.O. Box 2457, Riyadh 11451, Saudi Arabia

**Keywords:** consumer safety, herbal product, herbal spices, nutraceutical, plant-based medicine, traditional knowledge

## Abstract

Herbal spices are an agricultural commodity, economically very important and beneficial in primary healthcare in the food and medicine sectors. Herbal spices are used as food flavoring agents as well as in phytotherapies throughout the world and have nutritive benefits. The food and medicine industries widely employ artificial or natural adulteration to retard the deterioration and utilization of these adulterants in food and medicine products has given rise to significant apprehension among consumers, primarily stemming from the potential health risks that they pose. Thus, their characterization for the purpose of identification, origin, and quality assurance is mandatory for safe human consumption. Here, we studied 22 samples of commonly traded herbal spices that belong to 20 different genera and 21 species comprising 14 families, investigated macroscopically or organoleptically as well as histologically under microscopic examination. In this study, we provide details on organoleptic features including appearance, taste, odor, color, shape, size, fractures, types of trichomes, and the presence of lenticels among the examined herbal spices and these features have great significance in the detection of both natural as well as artificial deterioration. In terms of microscopic characterization, each examined plant part comprising different anatomical characteristics has taxonomic importance and also provides useful information for authentication from natural adulterants. Furthermore, the studied taxa were also described with nutritive and therapeutic properties. For condiments, herbal beverages and medicinal purposes, different herbal parts such as leaves, floral buds, seeds, fruit, and accessory parts like mericarp, rhizome, bulbs, and bark were used and commercially traded. Similarly, in this study, the leaves of *Cinnamomum tamala* and *Mentha spicata*, the floral buds of *Syzygium aromaticum*, the seeds of *Amomum subulatum*, *Brassica nigra*, *Punica granatum*, *Myristica fragrans*, *Phyllanthus emblica*, and *Elettaria cardamomum*, the mericarp of *Coriandrum sativum*, and *Cuminum cyminum* were observed. As a result, we show the potential of herbal spices as a source of many valuable phytochemicals and essential nutrients for food, nutraceutical, and homoeopathic medicine.

## 1. Introduction

Herbal spices are commonly cultivated for foodstuff, medicinal and economic value. They are either cultivated locally or imported from neighboring countries in dried or fresh parts, and used as flavor, color or preserved food in the culinary arts [1]. Herbs and spices have been used for health benefits for decades [2]. Nowadays, culinary herbs refer to plants with parts, like roots, stems, leaves, bark, flowers, floral buds, and stigma of the carpel, that are used as spices [3]. Herbal spices include the edible parts of plants that have rich sources of phytochemicals that are traditionally added to foodstuffs for their natural flavorings, aroma, visual appearance, pungency in dishes, and preservative purposes [4]. Some economically important and commonly traded herbal spices include rhizome (turmeric and ginger), buds (cloves), fruits (emblic), bark (cinnamon), leaves (malabar and mint), seeds (fenugreek and mustard) and mericarps of ajwain, fennel and cumin [2]. Furthermore, herbal spices have been used since ancient times not only to improve the flavor of foodstuffs but because they also have beneficial actions including antibacterial, anti-inflammatory, antioxidant, anti-hypertensive, anti-thrombotic, cardiovascular and chemopreventive effects to treat many metabolic health problems [5]. It is also estimated that several spices such as garlic, ginger, turmeric, cinnamon, pepper, and cardamom have a particular interest in their special modulatory effects on atherosclerosis, cancer, diabetes, obesity, arthritis, immune deficiency, ageing, and mental health [6,7]. Further, many spices like garlic, ginger, cumin, cinnamon, fennel, and mint are used in herbal beverages like herbal tea [8]. Thus, nowadays, different types of herbal spices such as Cumin, fennel, mint and ginger are used as superfoods containing high levels of nutrients [9]. The remarkable properties of these organic superfoods also include phytochemical compounds with lots of health benefits [10]. Hence, the high consumption of spices and culinary herbs has led to problems in their identification and authentication from deterioration. Deterioration is a genuine problem associated with commonly traded herbal spices [11]. So, histological examination is needed to confirm they are free from natural and artificial adulteration.

Anatomical features are the most important features for the identification and authentication of economic medicinal plants [12]. The anatomical features of plant organs cannot be neglected because these are considered the backbone in the studies of anatomy, cell biology, and taxonomy, which provide evidence for the delimitation of plant taxa [13]. Among the quality controls, the parameters for the characterization of medicinal plants recommended by World Health Organization (WHO) include organoleptic and microscopic evaluation [14,15,16]. The purpose of these characterizations is to ensure the authentication of spicy plants. As a result, histological studies can aid in the identification of herbal spices. The anatomical examination of medicinal plants involves the use of diagnostic tools for correct identification [17]. Studies of spices from an ethnobotanical point of view have shown that they have lots of uses in medicine other than food purposes. In the last two decades, research has been carried out on secondary metabolites in plants due to their widespread use and appreciation by both rural and indigenous peoples.

In developing countries like Pakistan, India, Sri Lanka, and Bangladesh, where herbal plants are cultivated for economic value, the commercialization of herbal materials based on the food and herb industries is dependent upon the availability of facilities and information associated with marketing the industrial potential of herbal plants [18]. Similarly, culinary and herbal spices are economically very important and are commonly traded within the country [3]. Medicinal plants of various remedies were exported from developing countries for economic revenue at the commercial level for cash income in a variety of forms, such as fresh, ripened, dried, broken, crushed, and powder forms [19]. Following that, herbal materials with active chemical constituents in their parts, such as rhizomes, dried leaves, fruits, barks, and bulbs, are recommended for the treatment of various ailments in humans and other animals [20]. From a nutraceutical standpoint, spices are very important in our daily food because they provide basic health benefits such as the prevention and treatment of various disorders [21].

Herbal spices are commonly used by the peoples of Pakistan and across the world and have great importance in the indigenous culinary and conventional systems of medicine. Pakistan has a huge market for economically important herbal raw materials for cultivation, processing, exportation, and consumption, which has lots of trouble with their identification and authentication. After India and Sri Lanka, Pakistan is also the main contributor to spice consumption and cultivation as well. The spices are used for their health significance [22]. Similarly, geographically, Pakistan provides a significant land trade route for the exportation of herbal raw materials from central Asian countries to India, China, Bangladesh, and Sri Lanka. Thus, the study area covers an area of 80,943 km^2^ and is located between 60°55′ and 75°30′ E longitude and 23°45′ and 36°50′ N latitude [23]. It has an altitudinal range from 0 to 8611 m, with a large area for cultivated and naturalized medicinal plant taxa [24]. The study area includes habitats ranging from the seashore to deserts, as well as high mountainous regions and plain agricultural land [25]. Furthermore, Pakistan has 6000 native species of higher plants, with 400 endemic taxa mostly restricted to the mountainous region [26,27]. Ethnobotanically, Pakistan hosts 600 to 700 medicinal plants, out of which more than 170 species have been recorded for spices and culinary purposes [28,29].

The objective of this study is to provide a detailed account of the economically important herbal spices with their nutritive and therapeutic uses. These herbal materials were evaluated both macroscopically and microscopically for their taxonomic significance. Organoleptography and histology provide valuable information for their correct identification and authentication from artificial and natural deterioration. In addition, the diagnostic features observed through this artistic work provide a roadmap for pharmacognostic standardization. Furthermore, histological analysis of the commonly traded spices will help in the improvement and quality control of these products. Additionally, these herbal spices are not only important for their food and nutritive values but also for their therapeutic potential.

## 2. Materials and Methods

### 2.1. Samples Collection Sites

**Figure 1:**
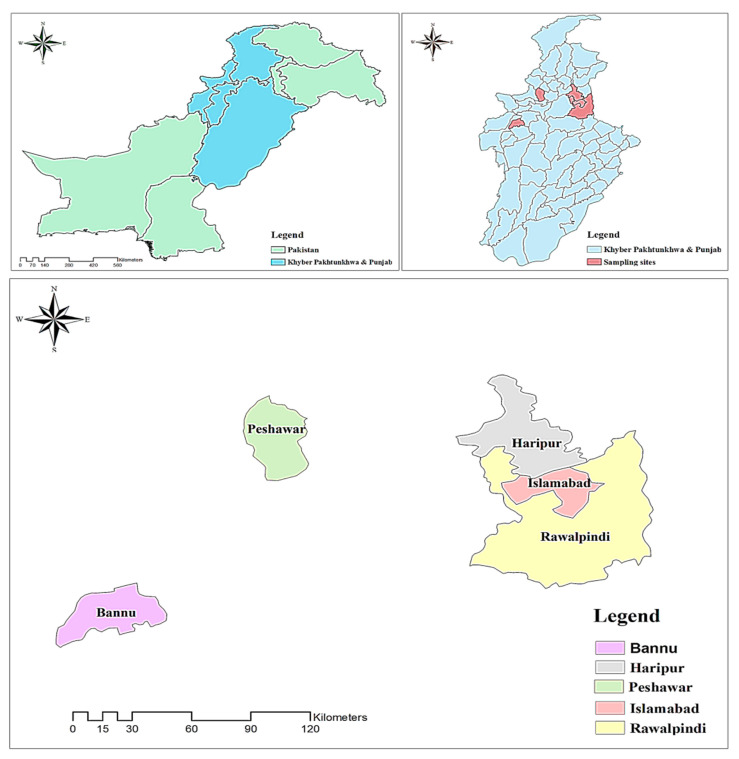
Map showing sampling sites in the investigated area.

All samples of the economically important herbal spices were collected from various sites, i.e., herbal trading markets, imported local herbal shops, local cultivation field crops and the food and medicine industries in the study area. The collection sites were selected based on their famous traditional spicy dishes and huge consumption. Study visits were conducted from January 2020 to April 2023 from various sites (Bannu, Peshawar, Haripur, Rawalpindi and Islamabad) as shown on the map of the study area (Figure 1). Interviews were also conducted with different respondents like local elders, farmers, herbalists, healers, local dealers, and traders to record the local name of the plant, traditional uses, parts used in food and medicine, parts traded, export materials, import remedies, and utilization. Therefore, each specimen was properly photographed and then identified by an expert herbalist (Medicinal plant taxonomist). For further identification, the specimens were confirmed by the histological features described in several published literature [16,21,30]. Further, all the investigated herbal materials were deposited to the herbarium of Quaid-i-Azam University, Islamabad (ISL), Pakistan with their proper voucher numbers. All the investigated plant materials underwent a meticulous verification process to ensure precision and conformity with established references, including World Flora Online Plant List (https://wfoplantlist.org/ (accessed on 4 April 2021)), World Flora Online (https://www.worldfloraonline.org/ (accessed on 21 August 2022)), the International Plant Name Index (IPNI) (www.ipni.org (accessed on 13 October 2022), and Plants of the World Online (https://powo.science.kew.org/ (accessed on 3 March 2023)). Thus, each species is mentioned with its synonym, spicy name, common name, as well as generic name, along with its respective family (Table 1 and Table 2).

### 2.2. Nutraceutical Survey of Spices

In the present study, from a nutraceutical standpoint, herbal spices are very significant in our daily food because they provide basic health benefits such as the prevention and treatment of various disorders [21]. Similarly, herbal spices were not only used as spicy materials but also had a nutritive and therapeutic potential as well in local communities [31]. Twenty-two samples of herbal spices were chosen for this study, with an emphasis on their nutritional and therapeutic properties [16,30]. Various hotels and spice markets were visited for the consumption of spices in food dishes such as salads, condiments, and herbal tea. In addition, questionnaires were devised by the condiment shopkeepers to obtain information about the uses of the investigated spices in various types of condiments in the study area. The consumption of spices other than food was also recorded by the various industrial sites and herbalists in different Pansar shops were chosen for their accessibility, ability to produce herbal products, diversity of plant parts, high utilization, and trade potential in the studied area.

### 2.3. Specimen’s Preservation

**Figure 2:**
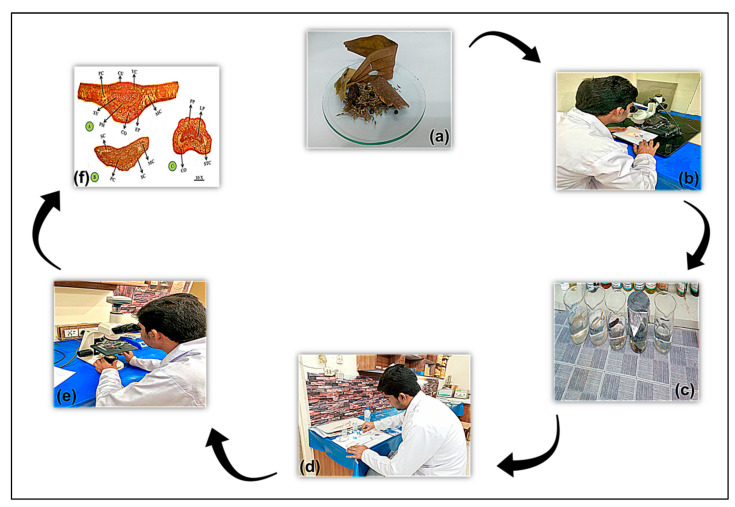
(**a**) Herbal spices, (**b**) macroscopic examination, (**c**) sample preparation, (**d**) slide preparation, (**e**) microscopic examination, and (**f**) histology.

The specimens were preserved in F.A.A solution (prepared by adding formaldehyde, acetic acid, and ethyl alcohol in 5:5:90 ratios, respectively) for two weeks, and then the specimens were ready for histological study in Figure 2, following the methodology of [32].

### 2.4. Histological Preparation

For histological examination, we have made a clean and thin transverse section of each specimen following the methodology of [21], with slight modification in which the specimens were washed and cleaned with distilled water and passed through a series of ethanol concentrations of 60, 80, and 90%, respectively for dehydration purpose. Then, the dehydrated specimens were penetrated by the molten paraffin wax at 75 °C. Specimens were kept in molten paraffin wax in the base mold, which was used for this purpose. The base mold, along with sections, was put in an ice bath for five to eight minutes to cool the wax and fix the specimens in the wax. Later on, the base mold was removed, and the specimens were ready for trimming and cross-sectioning. The thin and clear sections were taken by performing the Shandon Microtome (Finesse, 325) [33]. A thin transverse section was taken with a thickness between 30–45 µm through Microtome and manually. Further, the sections were treated with 50% chloral hydrate solution and then shifted on a hot plate and kept in the oven at 60 °C to allow the wax to expand at that temperature. Finally, the sections were washed with distilled water along with a bleaching solution and then treated with a different series of alcohol (60, 70, 80, and 90%) concentrations, respectively. Therefore, the dehydrated sections treated with 0.1% safranin O and 0.15% fast green stains were used for staining purposes. After staining, Canadian balsam was dropped on the slide and a cover slip was put on the section [13,21].

### 2.5. Macroscopic/Organoleptic Examination

For the organoleptic characters, protocols were followed using sensory organs and with the help of an Olympus stereoscopic microscope (Model, 605371) in Figure 2, [15,21,34]. We observed both internal and external macroscopic features, including color, taste, odor, shape, size, fractures, types of trichomes, and the presence of lenticels, in the studied samples followed by [35].

### 2.6. Microscopic/Anatomical Examination

The prepared slides were examined under light microscope Model (MEIJI Techno MT4300H) with a 10 to 40× objective in Figure 2. Micrographic images of each specimen were taken with a digital camera Model (MEIJI Techno; HD1500T) fitted with a light microscope at 10 to 40×. Furthermore, the images were properly labelled in Adobe Photoshop (Version: CS 8.0). Later on, descriptive terminologies for anatomical features were referenced from the standard anatomy glossary [13,16,21,30,36,37].

## 3. Results

We have studied 22 economically important herbal spice specimens from 21 different plant taxa that belong to 20 genera and 14 families. They were studied macroscopically, microscopically, and with their uses in herbal tea, condiments and therapeutics. Consequently, different plant parts such as bark (cinnamon), bulb (garlic), floral buds (clove), fruits (chili, anise star, black pepper and emblic), fruit peel (mace), leaves (malabar leaf and mint), rhizomes (ginger and turmeric) and seeds (greater cardamom, mustard, cardamom, pomegranate, nutmeg and fenugreek seeds) and mericarps (coriander, cumin, carom, and fennel) were observed macroscopically and histologically. Also, the therapeutic and nutritive properties are summarized in Table 3. Details of each specimen are described below.

### 3.1. Macroscopic and Microscopic Characterization

Macroscopically, the studied specimens were observed through sensory organs and by using a stereomicroscope. Similarly, for further examination, the investigated taxa were examined under light microscopy. The organoleptic as well as histological features of each plant part are given. 

***Allium sativum*** L. 

**Macroscopic features:** Garlic is a bulbous perennial herb. It has whitish scaly, papery leaves, a light yellowish internal color, a glossy surface, fractured flesh, a pungent odor, and a bitter taste that warms the tongue.

**Figure 3:**
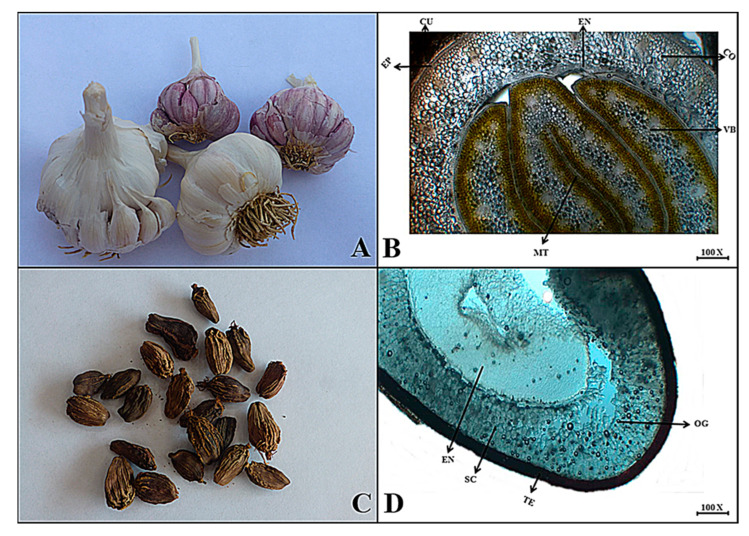
(**A**) Micrograph of garlic bulb. (**B**) Transverse section of *Allium sativum* bulb: Cu—cuticle, Ep—epidermis, Co—cortex, En—endodermis, Vb—vascular bundles and Mt—mesophyll tissues. (**C**) Micrograph of greater cardamom. (**D**) Transverse section of *Amomum subulatum* fruit: Te—Testa, Sc—sclerenchymatous region, Og—oil globules and En—endospermic region.

**Microscopic features:** The transverse section of the garlic bulb shows that the outermost blackish to brown cuticle, followed by a single layer of thin-walled cells, forms an epidermis. Several layers of spherical to oval-shaped cells lie below the epidermis considered a cortical region, the cortex projects a single layer of cells inside to the center as an endodermis. A few clusters of vascular bundles lie in center of the bulb. Finally, several coiled layers of mesophyll tissues are present in the middle region of the bulb as shown in Figure 3.

***Amomum subulatum*** Roxb. 

**Macroscopic features:** Greater cardamom fruit is a capsule, mostly long ellipsoid to slightly curved, flat on one side, with three distinct locules and approximately 15 to 20 seeds in each locule. The upper surface is considered a pericarp, grayish-brown to brown with longitudinally winged ribs making the surface wrinkled. A seed of greater cardamom is irregularly ovoid to polyhedral in shape, externally blackish brown, with a colorless, membranous aril, whitish greyish internal color, smooth to wrinkled surface, brittle fracture, aromatic odor and slightly pungent taste.

**Microscopic features:** In the transverse view of greater cardamom, the upper layer consists of epidermal testa with relatively thickened wall cells providing brown to red pigment along with oil cells. The sclerenchymatous region consists of sclerenchyma cells and also has some spherical to oval-shaped oil globules. The central region consists of a large lumen surrounding the small thickened walls and a sclerenchymatous layer as shown in Figure 3. 

***Brassica nigra*** (L.) K. Koch 

**Macroscopic features:** Black mustard seeds are generally round to oval in shape, reddish to yellowish in color, internally brown, with a smooth surface, brittle fracture, specific odor and slightly bitter taste. 

**Figure 4:**
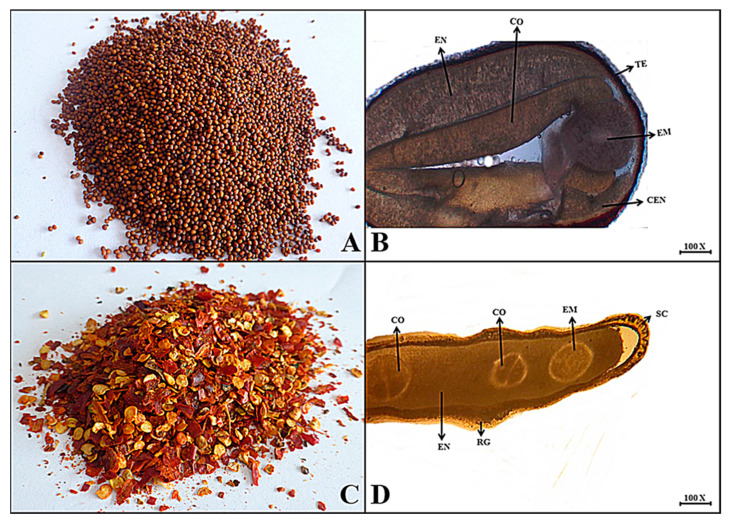
(**A**) Micrograph of mustard seed. (**B**) Transverse section of *Brassica nigra* seed: Te—testa, En—endosperm, Co—cotyledon, Em—embryo and Cen—chalazal endosperm. (**C**) Micrograph of pepper seed. (**D**) Transverse section of *Capsicum annuum* Seed: Sc—seed coat, Rg—ridges, Co—separately localized cotyledons, En—endosperm and Em—embryo.

**Microscopic features:** The transverse section of mustard seed has an upper-most epidermal testa layer followed by the endospermic region consisting of thick walled sclerenchymatous cells. Further, two cotyledons surrounding the embryo at the end coiled form chalazal endosperm were examined (Figure 4).

***Capsicum annuum*** L. 

**Macroscopic features:** Chili pepper crushed fruit is used as a spice. It has reddish to brown pieces with yellow seeds, an internal light yellow color, wrinkled surface, brittle fracture, and highly pungent odor. Its taste is highly bitter.

**Microscopic features:** A transverse section of chili seed shows that the outer hard layer consists of thick-walled sclerenchymatous cells, making a seed coat with the projection of several ridges. The cotyledons were separately localized in the middle region, and the embryo was embedded in the endospermic region at the polar end of the seed, as shown in Figure 4. 

***Cinnamomum tamala*** (Buch.-Ham.) T. Nees & Eberm. 

**Macroscopic features:** Bay leaves are used in dry conditions in different condiments; they have an ovate, oblong to lanceolate shape and appearance, are brown in color, internally light brown in color, with a smooth surface, soft fracture, aromatic odor, and slightly sweetish taste.

**Figure 5:**
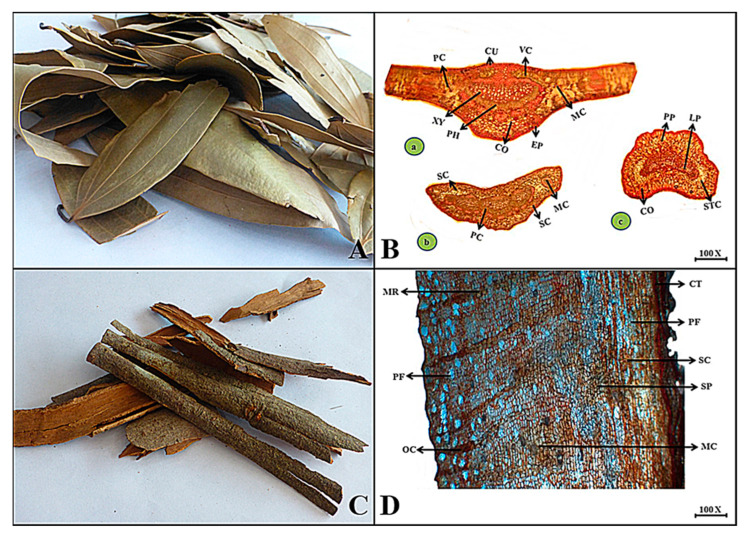
(**A**) Micrograph of malabar leaf. (**B**) Transverse section of *Cinnamomum tamala* leaf (**a**) leaf lamina, (**b**) leaf base and (**c**) leaf petiole: (**b**,**c**) Cu—cuticle, Ep—epidermis, Mc—mucilage cells, Vc—volatile oil cavity, Lp—lignified parenchyma, Pp—pitted parenchyma, Sc—sclerenchyma, Co—collenchyma, Pc—pericycle, Mc—mesophyll cell, Pc—palisade, Xy—xylem, Mc—mucilage cavity, Ph—phloem, Stc—stone cell with tannin and Sc—sclerenchyma. (**C**) Micrograph of cinnamon bark. (**D**) Transverse section of *Cinnamomum verum* bark: Ct—cork tissues, Pf—pericyclic fibers, Sc—sclereids, Sp—secondary phloem, Mr—medullary ray, Mc—mucilage cells, Pf—phloem fibers and Oc—oil cavity.

**Microscopic features:** Microscopically, in malabar leaf, the uppermost cuticle layer is followed by a single layer consisting of thin-walled cells as an epidermis at the lower and upper surfaces. The sclerenchymatous region consists of mucilage cell cavities and volatile oil cavities, followed by lignified parenchyma along with pitted parenchyma. The middle portion consists of a pericycle that includes xylem and phloem with stone as well as tannin. The mesophyll cells, palisade parenchyma, and lower cortex covered the vascular bundle in the midrib as seen in Figure 5.

***Cinnamomum verum*** J. Presl

**Macroscopic features:** Cinnamon inner bark is half-folded and dull to brown in appearance. The internal color varies from dark brown to light brown. The surface is rough due to fibers, brittle to splintery, and the odor is fragrant and sweetish, giving a warming sensation to the tongue.

**Microscopic features:** In a transverse view of the inner bark of cinnamomum, the upper portion consists of loose cells making the cork tissue followed by pericycle fibers consisting of longitudinally squared cells with brown to red pigment. Sclereids and secondary phloem lie in a parenchymatous zone merged with tangentially elongated medullary rays with mucilaginous cavities. The phloem fibers also consist of deep channels for oil cavities, as shown in Figure 5. 

***Coriandrum sativum*** L. 

**Macroscopic features:** Coriander has a dried mericarp that is brownish to yellow in color, oval to oblong in shape, externally brown, slightly rough surface due to small ridges, soft fracture, specific aromatic odor and spicy taste.

**Figure 6:**
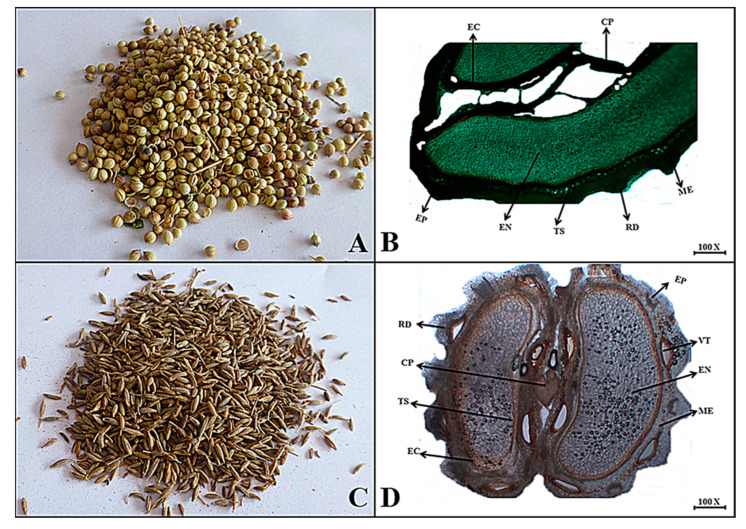
(**A**) Micrograph of coriander mericarp. (**B**) Transverse section of *Coriandrum sativum* mericarp: Rd-ridges, Ep—epicarp, Me—mesocarp, Ec—endocarp, Cp—carpophore, Ts—testa and En—endosperm. (**C**) Micrograph of cumin mericarp. (**D**) Transverse section of *Cuminum cyminum* mericarp: Rd—ridges, Ep—epicarp, Vt—vittae, Me—mesocarp, Ec—endocarp, Ts—testa, Cp—carpophore and En—endosperm.

**Microscopic features:** The transverse section of the coriander mericarp consists of hard testa with a single layer of epicarp along with several ridges followed by thick-walled sclerenchymatous cells making up the endocarp region of the mericarp. Mostly, the endocarp consists of endospermic thin-walled parenchymatous cells and, finally, the carpophore, the stalk that deeply penetrates the entire mericarp as shown in Figure 6. 

***Cuminum cyminum*** L. 

**Macroscopic features:** Cumin has capsule-shaped dark brown mericarps, with a whitish to brown internal color, rough surface due to ridges, brittle fracture, specific odor and spicy taste.

**Microscopic features:** Transversally, the cumin mericarp consists of the outer layer testa with the epicarp region along with several irregularly shaped ridges, including brown fragments of vittae, which are composed of thin-walled cells. The mesocarp consists of several layers of reticulate parenchyma cells with thick, lignified walls with oval to rounded pits, while the endocarp is composed of thin-walled, lignified cells of endosperm arranged in groups parallel to one another, as seen in Figure 6.

***Curcuma longa*** L. 

**Macroscopic features:** Turmeric rhizome is slightly cylindrical with small appendages and black to brown papery bract leaves, a dark-yellow internal color, rough surface due to scaly and bract leaves ridges, hard fracture, pungent odor and astringent taste. 

**Figure 7:**
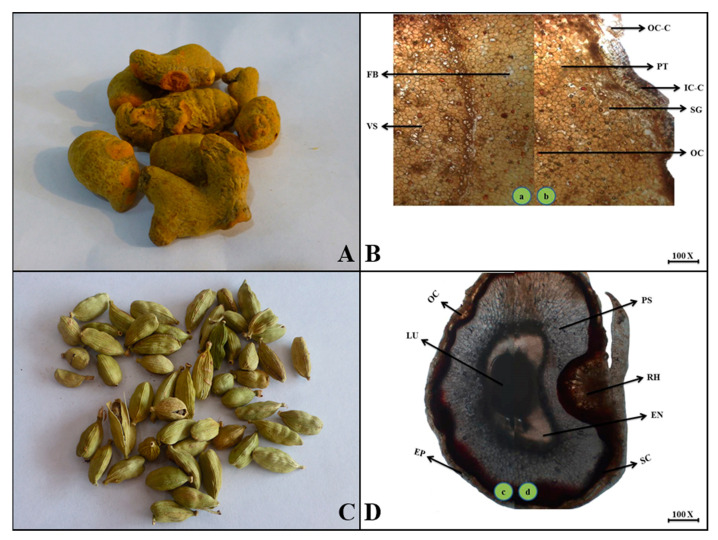
(**A**) Micrograph of turmeric rhizome. (**B**) Transverse section of *Curcuma longa* rhizome: (**a**) Fb—fibers and Vs—vessels. (**b**) Oc.c—outer cork cells, Pt—parenchymatous tissue, Ic.c—inner cork cells, Sg—starch grains and Oc—oleoresin cells. (**C**) Micrograph of cardamom fruit. (**D**) Transverse section of *Elettaria cardamomum* fruit: (**c**) Ep—epidermis, Lu—lumen and Oc—oil cellular layer (**d**) Sc—sclerenchymatous layer, En—endosperm, Rh—raphe and Ps—palisade sclerenchyma.

**Microscopic features:** In this microscopic view of the turmeric rhizome, the outer layer composed of loosely packed fragments of cork is seen to make an outer cork, but the inner cork tissue consists of closely packed fragments of cork cells with pale brown, thin walls, and striated. Parenchymatous tissues are observed abundantly in small groups with gelatinized scattered starch grains and bright to yellow-colored oleoresin cells. On clearing, phloem fibers and xylem vessels are also composed of round to oval cells with thin and irregular walls, scattered in the ground tissue as shown in Figure 7. 

***Elettaria cardamomum*** (L.) Maton.

**Macroscopic features**: True cardamom fruit is a capsule, slightly long to ellipsoid, with three obtuse ridges, each having three locules with two to seven seeds in each locule. The upper surface is considered a pericarp, bluish green to yellowish green in appearance, with longitudinal furrows and ribs arranged densely. Dehiscent takes place from the base. The seeds are protected by a colorless membranous aril. Due to striations, the surface of the seeds is smooth to rough, brittle fracture, aromatic, and slightly bitter to pungent in taste.

**Microscopic features:** In the transverse view of the capsule, it consists of a single layer of epidermal testa with longitudinally elongated cells followed by several layers of sclerenchymatous cells consisting of an oil cellular layer with brown-red pigment that makes up the hypodermis layer. In the mid region, a tangentially elongated raphe region was observed. The palisade sclerenchymatous region has brown cells with thick walls that surround the endospermic portion around the lumen as shown in Figure 7. 

***Foeniculum vulgare*** Mill. 

**Macroscopic features:** Fennel mericarps are oblong to ovoid in shape, greenish in appearance, brown to whitish internally, rough surface due to ridges, brittle fracture, and aromatic and sweetish in taste. 

**Figure 8:**
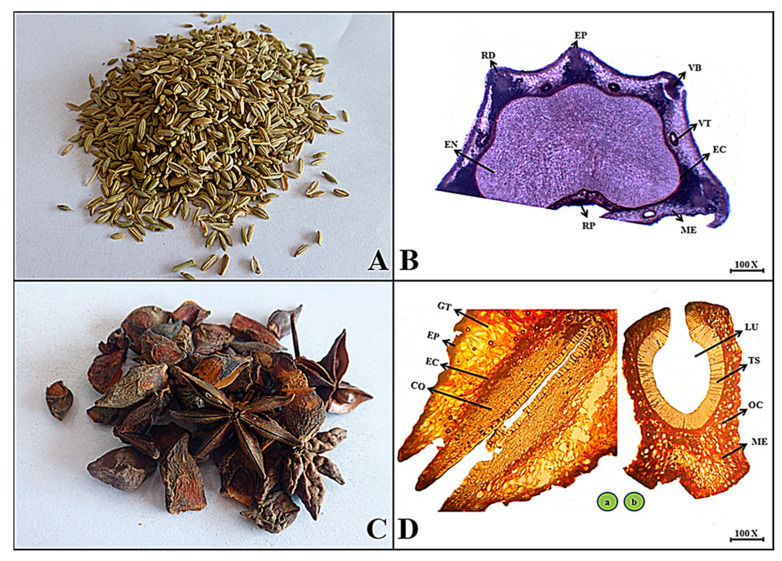
(**A**) Micrograph of fennel mericarp. (**B**) Transverse section of *Foeniculum vulgare* mericarp: Rd—ridges, Ep—epicarp, Vt—vittae, Rp—raphe, Me—mesocarp, Ec—endocarp, Vb—vascular bundle and En—endosperm. (**C**) Micrograph of star anise fruit. (**D**) Transverse section of *Illicium verum* fruit: (**a**) Ep—epicarp, Gt—ground tissue, Ec—endocarp region, and Co—cotyledon. (**b**) Ts—testa, Oc—oil cavity Lu—lumen and Me—mesocarp region.

**Microscopic features:** In a microscopic view of fennel, the mericarp shows an outer pigmented layer composed of thin-walled cells that are polygonal in shape, forming an epicarp that projects to several ridges and is composed of some irregularly shaped vittae and a deep furrow in the mid region as a raphe contains reticulate parenchyma with ovoid to elongated lignified walled cells. Many vascular bundles are scattered throughout the mesocarp and endocarp, but the central region containing endosperm fragments is composed of polygonal thick-walled cells with fixed oil globules and aleurone grains, as shown in Figure 8. 

***Illicium verum*** Hook. f. 

**Macroscopic features:** Star anise fruits are blackish to brown in color with a star shape in appearance, an internal color of reddish to dark brown, slightly rough surface due to the presence of wrinkles, brittle fracture, pleasant scent, and intensely spicy-sweet to pungent taste.

**Microscopic features:** The transverse section shows the upper layer composed of thick-walled cells with pitted lignified cells of the epicarp region followed by ground tissue including many oil cavities scattered in pigmented, polygonal-shaped parenchymatous tissues that make up the mesocarp. Secondly, the endocarp region contains seed features with hard, pitted lignified cells that surround the large lumen and provide seeds. The seed cross-section consists of a seed coat with hard and thick-walled cells followed by two long, straight, and parallel cotyledons as seen in Figure 8. 

***Myristica fragrans*** Houtt. 

**Macroscopic features:** Nutmeg dried seeds are spherical to oval with a brownish appearance, light brown to dark brown internal color, rough surface due to ridges and furrowed, brittle to short fracture, spicy odor and slightly warm taste. The fruit peel of mace is crimson-reddish to orange in color, with a thread-like appearance, an internal color of orange to brown, a smooth surface, a soft to brittle fracture, a pleasant and spicy odor, and a sharp to warm taste.

**Figure 9:**
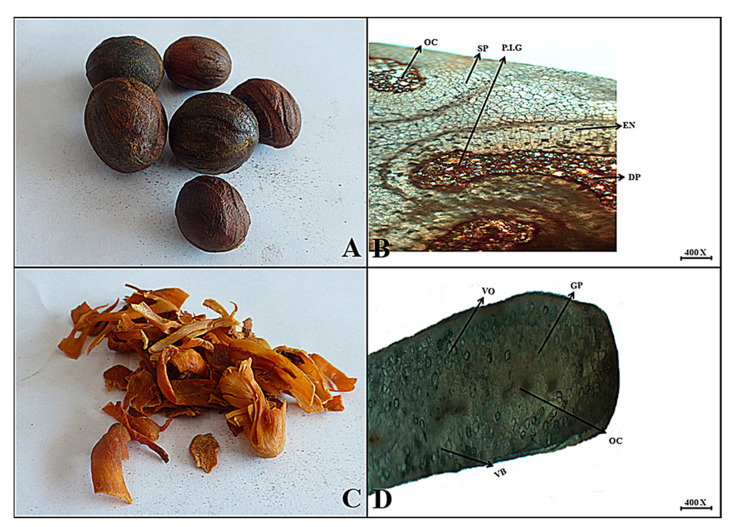
(**A**) Micrograph of mace seed endosperm. (**B**) Transverse section of *Myristica fragrans* seed endosperm: Sp—spongy parenchyma, Dp—dilated parenchyma, Oc—oil cavity, En—endosperm and Pig—perisperm inner growth. (**C**) Micrograph of fruit peel of Mace. (**D**) Cross-section of fruit peel of *Myristica fragrans*: Vb—vascular bundle, Gp—ground parenchyma, Oc—oil cavity, and Vo—volatile oil contents.

**Microscopic features:** A transverse section of the Mace seed endosperm reveals an oval to polygonal shape, spongy parenchyma cells, and dilated parenchyma. Furthermore, oil cavities were embedded in the endospermic as well as perisperm regions. Similarly, in a transverse view, the aril is flat and isobilateral, but it is made up of many irregular vascular bundles scattered throughout the ground parenchyma, each with distinct oil cavities and varying volatile oil contents (Figure 9). 

***Mentha spicata*** L. 

**Macroscopic features:** Spearmint crushed leaves and their young twigs are greenish in color, their internal color is greenish to brown in color, their surface is slightly rough due to small hairs like trichomes, and their odor is pleasant and spicy.

**Figure 10:**
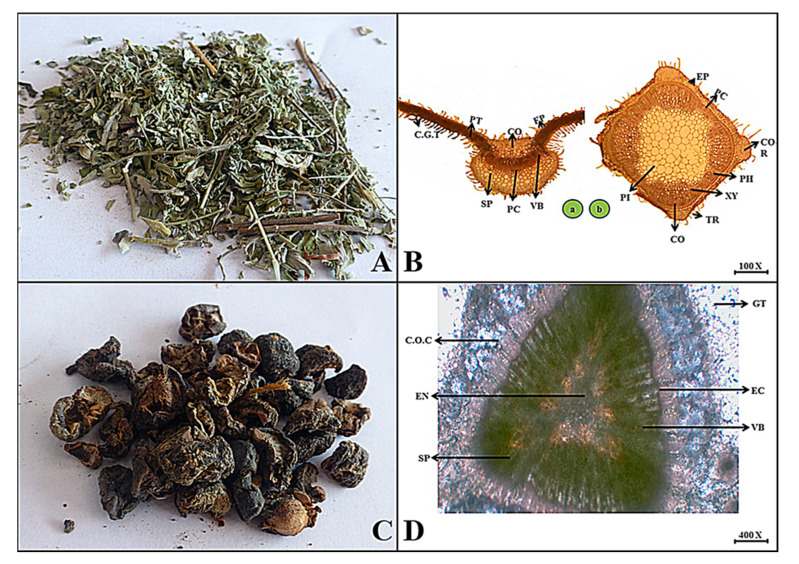
(**A**) Micrograph of spearmint leaf and young twig. (**B**) Transverse section of *Mentha spicata* leaf and young twig: (**a**) Ep—epidermis, Cgt—capitate glandular trichomes, Co—collenchyma cells, Pt—palisade tissues, Vb—vascular bundle, Pc—pericycle and Sp—spongy parenchyma. (**b**) Ep—epidermis, Cor—collenchymatous ridges, Co—cortex, Pc—pericycle, Ph—phloem, Xy—xylem, Tr—trichomes and Pi—pith. (**C**) Micrograph of emblic fruit. (**D**) Transverse section of *Phyllanthus emblica* fruit: Gt—ground tissue, C.o.c—calcium oxalate crystals, Sp—spongy parenchyma, En—endosperm, Ec—endocarp region and Vb—vascular bundle.

**Microscopic features:** In the transverse view of the young twig of Mentha, the uppermost single layer of cells as an epidermis with embedded trichomes is followed by several layers of sclerenchymatous cortical region with four angular collenchymatous ridges at each corner of the twig. A circular row of cells surrounds the vascular tissues, including the xylem and phloem. The center portion consists of an isodiametric oval in shape that contains a pith region. Similarly, the transverse section of the leaf consists of an epidermis containing a single layer of thin-walled cells along with capitate glandular trichomes followed by collenchyma composed of thick-walled and oval to polygonal cells. Palisade tissues laterally lie in a cup shape. The pericycle occupied the vascular bundles in the center of the midrib of the leaf lamina. There are several layers of spongy parenchyma cells below the vascular region. All the observed parts are covered with capitate glandular trichomes as shown in Figure 10. 

***Phyllanthus emblica*** L. 

**Macroscopic features:** The fruit of emblic is blackish to brown with six vertical furrows, the internal color is brown, the surface is crystalline and wrinkled in dry form, the fracture is brittle, the odor is distinct, and the taste is spicy sour.

**Microscopic features:** The transverse section of the dried fruit of emblic shows ground tissues consisting of calcium oxalate crystals with spongy parenchyma, but the endocarp has many small, irregular in shape, vascular bundles scattered throughout, as seen in Figure 10. 

***Piper nigrum*** L. 

**Macroscopic features:** Black pepper fruits are blackish in color with a round to oval shape, internal color of greyish to brown, and a surface that is rough due to wrinkled, fractured hardness. The odor is aromatic and spicy with a pungent taste. 

**Figure 11:**
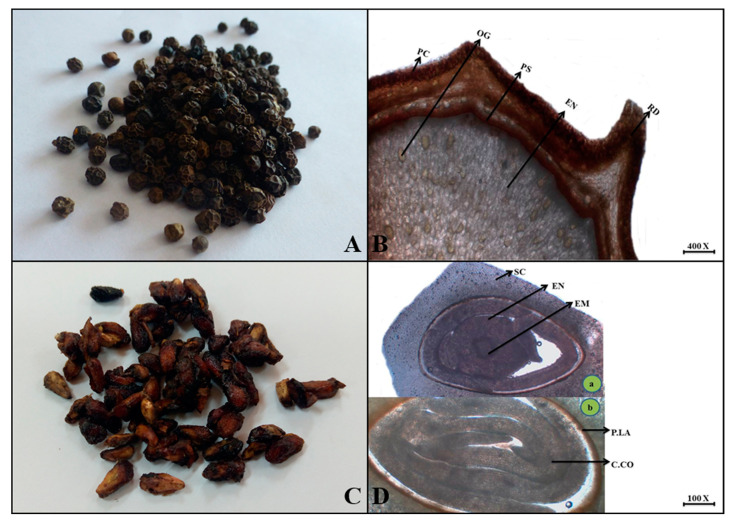
(**A**) Micrograph of black pepper fruit. (**B**) Transverse section of *Piper nigrum* fruit: Pc—pericap, Ps—perisperm, Rd—ridges, Og—oil globules, and En—endosperm. (**C**) Micrograph of Pomegranate seed. (**D**) Transverse section of *Punica granatum* seed: (**a**) Sc—seed coat, En—endosperm and Em—embryo. (**b**) P.La—pigmented layer and C.co—coiled cotyledons.

**Microscopic features:** In this transverse view of black pepper, the uppermost layer is a pericarp consisting of thick-walled parenchyma cells with curved ridges. But the inner portion is composed of an oval to sub-oval, thin-walled endospermic region with a polygonal shape and many yellowish oil globules as seen in Figure 11. 

***Punica granatum*** L. 

**Macroscopic features:** Pomegranate seeds are oval to oblong in shape, reddish-brown in color, grey to brown internal color, wrinkled surface, hard fracture, and have a pleasant odor and sour taste.

**Microscopic features:** Transversally, the seed of pomegranate has an outermost hard layer of thick-walled sclerenchyma cells that provide several ridges. A thin pigmented layer lies below the seed coat, followed by coiled cotyledons. The central portion occupies the endosperm and embryo. Thus, the endosperm is composed of thin-walled polygonal parenchyma cells with aleurone grains and fixed oil as shown in Figure 11. 

***Syzygium aromaticum*** (L.) Merr. & L. M. Perry 

**Macroscopic features:** Clove flower buds are unopened blackish to brown in color and nailed in shape, with an internal color of dusty reddish to dark brown, a smooth surface, brittle to hard fracture, pleasant aroma, and a spicy, pungent, and slightly tingling taste.

**Figure 12:**
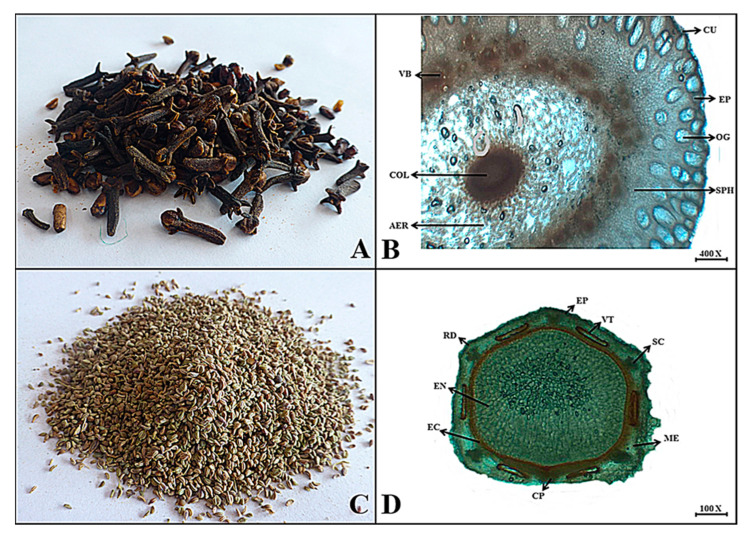
(**A**) Micrograph of Clove flower bud. (**B**) Transverse section of *Syzygium aromaticum* flower bud: CU—cuticle, EP—epidermis, OG—oil glands, COL—columella, SPH—sphaeraphide, VB—vascular bundles and AER—aerenchyma. (**C**) Micrograph of Carom Mericarp. (**D**) Transverse section of *Trachyspermum ammi* mericarp: Rd—ridges, Ep—epicarp, VT—vittae, SC—secretory cells, ME—mesocarp, EC—endocarp, CP—carpophore and EN—endosperm.

**Microscopic features:** Transactionally, the floral bud has an uppermost thick layer as a cuticle, followed by an epidermis consisting of a single layer of cells. The mid region consists of parenchymatous tissues and spheroidal cells with many vascular bundles arranged in a circular row surrounded by the aerenchyma with air spaces and has a central rigid columella as seen in Figure 12. 

***Trachyspermum ammi*** (L.) Sprague 

**Macroscopic features:** Ajwain mericarps are oval to conical with a greyish brown appearance, brownish internal color, rough surface due to prominent ridges, soft fracture, thymotic aromatic odor, and a spicy bitter taste.

**Microscopic features:** The transverse section of the mericarp consists of five concave-shaped ridges, with the epicarp composed of thick-walled and pigmented parenchyma. Several long and brownish-colored vittae are embedded in the mesocarp region along with secretory tissues. The innermost endocarp region consists of a central endospermic portion with thin-walled parenchyma cells as well as scattered oil globules and a brown to blackish rod-like structure, as shown in Figure 12. 

***Trigonella foenum-graecum*** L. 

**Macroscopic features:** Fenugreek seeds are deep yellow to olive colored, cuboid in shape, with a yellowish internal color, smooth surface, hard fracture, and odor like distinct maple syrup and slightly bitter taste.

**Figure 13:**
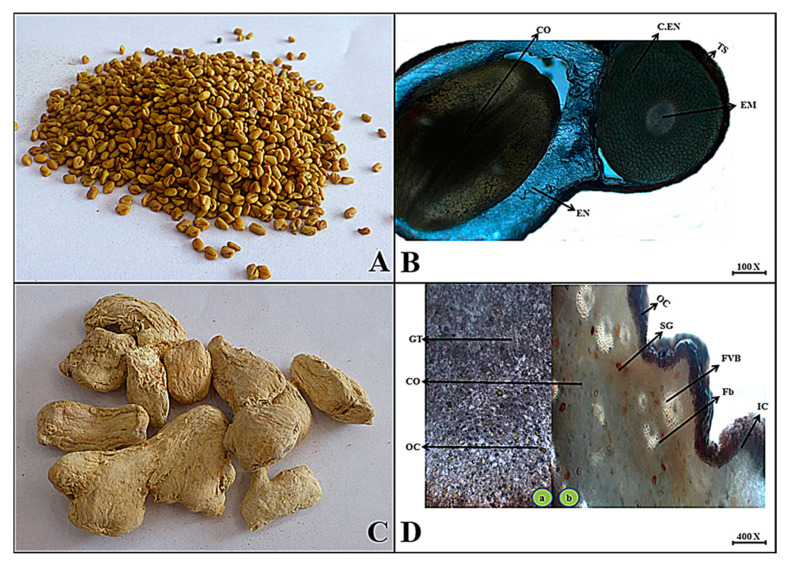
(**A**) Micrograph of fenugreek seed. (**B**) Transverse section of *Trigonella foenum-graecum* seed: Ts—testa, En—endosperm, Cen—chalazal endosperm, Em—embryo and Co—both the cotyledon. (**C**) Micrograph of ginger rhizome. (**D**) Transverse section of *Zingiber officinale* rhizome: (**a**,**b**) Oc—outer cork Co—cortex and Gt—ground tissues (**b**) Oc—outer cork, Ic—inner cork, Co—cortex, Sg—starch grains, Fvb—fibro—vascular bundles, Fb—fibers and Oc—oleoresin cells.

**Microscopic features:** The transverse section of fenugreek seed consists of the uppermost layers with thick parenchyma cells that make testa, followed by a whitish to creamy-colored region consisting of thin-walled parenchyma that makes an endosperm with two dumbbell-shaped cotyledons, and spherical to oval-shaped cotyledons. But spherical chalazal endosperm was also observed in the polar region consisting of oval to tubular thin-walled parenchyma cells along with the small central oval-shaped embryo as shown in Figure 13. 

***Zingiber officinale*** Roscoe 

**Macroscopic features:** Ginger rhizome has a pale-yellow appearance, an internal color of off-white to yellowish, a smooth surface, fibrous to hard fracture, a pleasant odor, and a spicy pungent taste.

**Microscopic features:** In the cross-sectional view of ginger rhizome, the outer and inner layer are made up of pigmented and colorless parenchymatous cells, followed by a cortex consisting of thick-walled parenchyma cells, but the ground tissues are composed of thin-walled parenchyma with starch grains, and many small-sized fibro-vascular bundles along with fibers as well as oleoresin cells scattered in the ground region as seen in Figure 13. 

### 3.2. Therapeutic and Nutritive Benefits of Spices 

**Figure 14:**
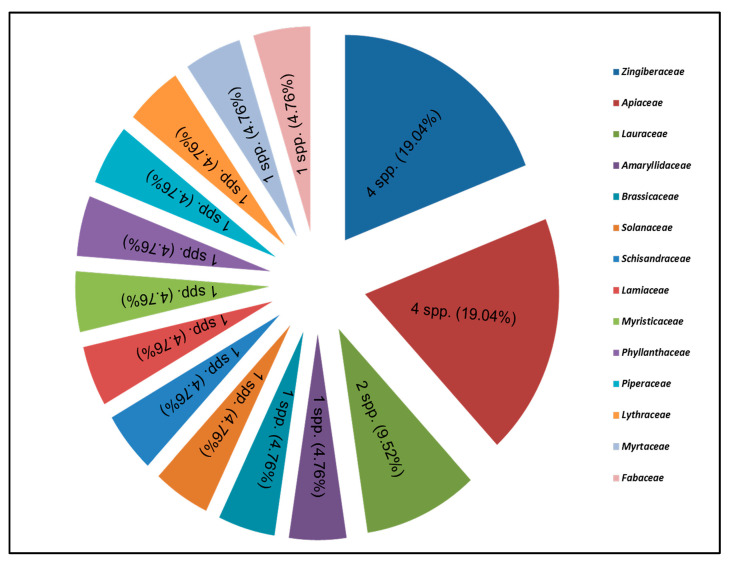
Representative plant families (numbers of taxa and its percentage).

Twenty-two herbal spices were chosen for this study, and different plant parts were used as nutritive and folk medicine supplements. As seen in Figure 14, the Apiaceae and Zingiberaceae families were the most numerous, with each having four species, but the Lauraceae family had two taxa, while the rest of the families only had one species. Food and spice markets, homoeopathic shops, and the herbal food and medicine industries in Rawalpindi, Islamabad, Peshawar, Haripur, and Bannu were visited to collect all of the information about their nutritive and therapeutic properties.

From ancient times, a number of herbal spices have been used for nutritive purposes. They are also used to prevent and treat many health issues; they are administered as an alternative therapy. In the modern era, cinnamon bark, ginger, true cardamom, and spearmint leaves are used as flavoring remedies for a variety of herbal beverages such as green tea, black tea, white tea, mint tea, cinnamon tea, and oolong tea. Similarly, in culinary terms, green chili, leaves of coriander, spearmint, and garlic are the essential ingredients for different types of sauces, such as garlic sauce, mint sauce, and coriander sauce. Thus, fennel, coriander, ajwain, bay leaf, greater cardamom, turmeric, chili pepper, black pepper, clove, nutmeg, mace, star anise, fenugreek, and ginger are the main ingredients for various types of condiments such as garam masala, salad masala, biryani masala, and achar masala, which give flavoring and pungency to daily delicious food dishes. Some herbal spices like cumin, fennel, mint, and ginger are used as superfoods containing high levels of nutrients. These organic superfoods also contain phytochemical compounds with lots of health benefits. In addition to these remarkable properties, herbal spices have also been used for their many traditional therapeutic properties like anticancer, antitumor, anti-inflammatory, antioxidant, antipyretic, and antitoxic effects (Table 3). 

### 3.3. Common Traded Spices Parts and Their Routes

**Figure 15:**
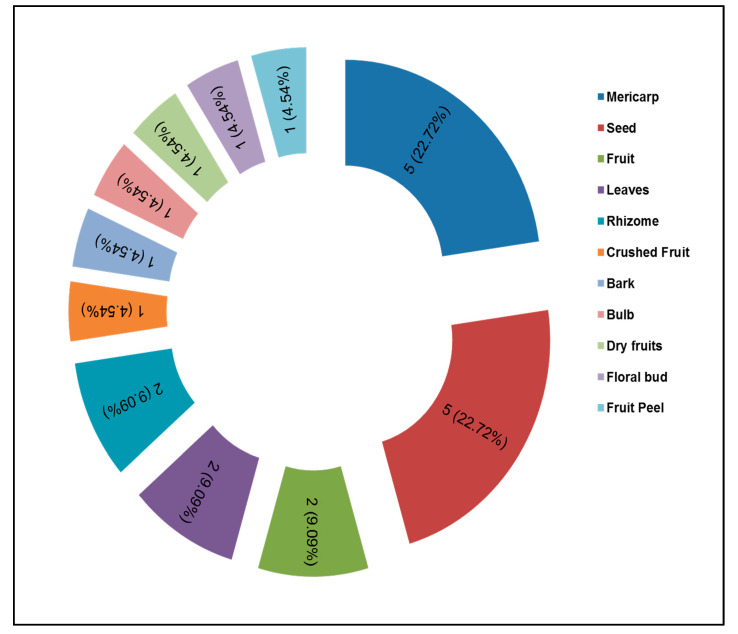
Representative plant parts traded (including frequency and its percentage).

In Pakistan, culinary and herbal spices are cultivated for economic purposes. However, the growth and processing of herbal spices provide cash income to the farmers and dealers, who are local exporters in the rural areas of the Punjab and Khyber Pakhtunkhwa. These spices are traded within the study area. But, due to their huge utilization in the food and medicine sectors, the local reserve stock will not be enough. The most traded spice parts were seeds, followed by fruit, leaves, roots, bark, and rhizomes (Figure 15). The trade routes were defined. Some spices were locally traded within the study area, while some were imported from neighboring countries like India, China, Iran, Afghanistan, Russia, and Sri Lanka. Similarly, many spices were exported, such as garlic, mint, turmeric, chili, mustard seed, pomegranate, fenugreek seed, cumin, carom, and fennel to South Asia, the Middle East, Malaysia, Singapore, Japan, Australia, the United Kingdom (UK), Germany, Holland, the United States of America (USA), and Canada. 

However, the examination of these herbal materials under macroscopic and microscopic tools is very important for the taxonomy of crude drugs. Macroscopic evaluation is considered the oldest method applied for crude drug identification and is still considered effective and accurate. Nowadays, histological features are not only used to identify the genuineness but to also improve the quality of herbal materials.

## 4. Discussion

People arrived from different countries and introduced their cultural culinary practices to the area [38]. Pakistan has a cultural heritage of traditional uses of spices in the food and medicine sectors from ancient times [39]. Nowadays, these products are economically very important and are easily adulterated by various means [11]. To gain a clear understanding of these products for future generations, their macroscopic and microscopic observations were undertaken.

The most economically important culinary herbs are traded in the spice and condiment markets and have a high utilization capacity at the industrial level for herbal products. Ninety two commonly traded herbal materials were reported from the various herbal markets of Rawalpindi District [40]. Similarly, 44 traded plant species were identified from the herbal markets of Makerwal and Gulla Khel. Thus, 103 herbal specimens were collected from different herbal markets in Khyber Pakhtunkhwa [38].

For the uses of culinary and herbal spices, 58 herbs were analyzed macroscopically in Nkonkobe Municipality, Eastern Cape, South Africa [1]. Similarly, for the authentication of herbal and medicinally important spices, organoleptic markers were applied as a good resolution in the detection of synthetic adulterants [41]. Thus, various methods were performed to determine the authenticity of common usage spices from Sri Lanka, India, and Bangladesh [19]. In China, physical and chemical, macroscopic and microscopic techniques were used to authenticate herbal materials [37]. Similar types of studies were also conducted on the macroscopic studies applied to the identification of Chinese herbal spices and medicinal plants [42]. Further, the quality of medicinally important herbs and spices was improved through a macroscopic examination [43].

Earlier studies were conducted on the herbal spices, including black pepper, caraway, cinnamon, cow parsnip, curry powder, garlic powder, red pepper, sumac, and turmeric, collected from the retail shop in Tehran, Iran and observed through a microbiological characterization [44]. Also, 27 different types of culinary spices affected by yeast were also collected from various markets in India and examined microscopically [45]. Similar studies were also carried out by various investigators on Indian spices such as pepper, turmeric, chili, and coriander, which were examined through gamma irradiation for quality improvement [46,47]. Some common condimental spices, e.g., black pepper, coriander, paprika, mace, pimento, and white pepper, were investigated in South Africa [48]. The quality of the contaminated spices was assessed through microscopic characterization in Botucatu, So Paulo, Brazil [49]. Four locally available spices, saffron (*Crocus sativus*), nutmeg (*Myristica fragrans*), mace (*Myristica fragrans*) and Shahi Jeera (*Bunium bulbocastanum*), were macroscopically and microscopically investigated in Dhaka, Bangladesh [50]. Similarly, 15 packed and 27 unpacked spice samples of three types (red pepper, turmeric and coriander) were also collected from different herbal markets in Bangladesh and examined for detection of microbial contamination [51]. Highly traded spices (black pepper) were improved through microscopic methods from adulteration by the addition of foreign berries and seeds or artificially prepared pepper from wheat or leguminous flour [52]. Thus, powdered black peppers were also detected through microscopic observation from common adulterated materials like pig meal, seed capsules of various fruits, stones, kernels, flowers, and minerals [53]. 

The bark of Ficus species, namely, *F. racemosa*, *F. virens*, *F. religiosa*, and *F. benghalensis*, is considered the most important ingredient of traditional medicine and was histologically examined [54]. An important, indigenous medicinal plant, *Fumaria indica*, was examined microscopically as well as physiochemically [55]. Thus, *Rhus succedanea* was also analyzed through histological, micromorphological, and physiochemical deterioration [56]. Other detailed studies also suggest the correct identification by using various microscopic techniques for its authentication [57]. Similar studies were also carried out on the crude drugs, which were examined by using various microscopical as well as macroscopical features [58]. A histological study was performed on the seed and mace of the nutmeg fruit, which is the leading spice in the food industry for quality control [59]. Therefore, the correct identification of medicinal plants was examined through histological observation to ensure the reproducible quality of phytomedicine [60]. Some traded spices and herbs were separated from artificial deterioration using analytical tools [11]. Hence, DNA barcoding techniques were performed for the authentication and genuineness of economic medicinal taxa of Lamiaceae from Pakistan and Fabaceae from China to detect adulteration or contamination [61,62].

### Nutritive and Therapeutic Potentials of Herbal Spices

Herbal spices are economically very important due to their huge consumption in food as well as in herbal products. Herbal spices are not only used as food but also have a high therapeutic potential. Traditionally, herbal spices have great importance in different systems of homoeopathic medicine. Similarly, many herbal industries use herbal spices as crude drug remedies in their homoeopathic products. In Pakistan, there are 105 herbal manufacturing companies that use herbs as ingredients. *Allium sativum* is therapeutically very important, including cardioprotective, inti-inflammatory, neuroprotective, anticarcinogenic, antimutagenic, and strong antidiabetic [63]. *Amomum subulatum* fruit extract has strong antibacterial and anti-inflammatory potential [64]. Mustard seeds exhibit significant anticarcinogenic properties against several types of cancer [65]. *Capsicum annum* has been reported to be antimicrobial, anti-inflammatory, cardioprotective, and beneficial to blood circulation [66]. Indian bay leaves have good chemical constituents that show important therapeutic properties, e.g., antidiarrheal, antitumor, anti-inflammatory, anti-arthritic, antiparasitic, and gastroprotective [67]. Cinnamon has been shown to have a variety of therapeutic properties, including antimicrobial, wound healing, antidiabetic, antiviral, anti-anxiety, and anti-Parkinson’s [68]. Coriander seeds were evaluated for their hepatic and renal protective potential [69]. Cumin seeds are traditionally applied to varieties of diseases like hypolipidemia, cancer, and diabetes [70]. Many pharmacological actions for Indian traditional spices such as *Punica granatum*, *Curcuma longa*, and *Zingiber officinale* have been reported, which have been suggested for the prevention of cancer and other chronic inflammation [71]. Fennel seeds were used in folk medicine for the treatment of obstructions of the liver, spleen, and gall bladder and for other gastro-intestinal complaints such as colic, indigestion, nausea, and flatulence [72]. *Illicium verum* is a traditional Asian spice widely used as a carminative, stomachic, stimulant and diuretic [73]. *Mentha spicata* has a wide range of therapeutic potentials, such as analgesic, anti-inflammatory, antipyretic, anti-androgenic, antimicrobial, antiviral, anticancer, antiemetic, and cytotoxic [74]. Nutmeg is a famous spice that exhibits numerous pharmacological activities such as antioxidants, anti-inflammatory, antifungal, antibacterial, and anti-diabetic properties [75]. The fruit pulp of emblic has antidiarrheal, anti-inflammatory, anti-diabetic, hypolipidemic, antibacterial, antioxidant, antiulcerogenic, hepato- and gastro-protective, and chemopreventive properties [76]. For black pepper, diverse therapeutic properties were reported, such as antihypertensive, antiplatelet, antitumor, analgesic, antiasthmatic, anti-inflammatory, antispasmodic, and anti-diarrheal [77]. Clove has been reported to have anticancer, antidiabetic, anti-inflammatory, and anti-protozoal properties [78]. Ajwain is a common spice and provides various pharmacological potentials like antioxidant, antifungal, antinociceptive, cytotoxic, antihypertensive, antispasmodic, diuretic, and anthelmintic [79]. Furthermore, fenugreek seeds are one of the oldest herbs that have been used to treat diabetes, hyperlipidemia, inflammation, and cancer [80,81].

Undoubtedly, herbal spices mixed with salt would have been used as preservatives for vegetables and pickles. Pungent spices such as chili, garlic, mustard seeds, black pepper, cardamom, clove, and ginger were effective in masking salty flavors in food dishes, aromatic spices such as cardamom, clove, spearmint, and nutmeg would be useful to disguise foul breath [82]. However, some spices, such as ginger, spearmint, and cinnamon, were used in herbal tea beverages at the domestic level for health purposes ranging from digestive complaints to aphrodisiac therapies. Pakistan still produces hundreds of tonics from various spices and herbs, and they are marketed as cough and gastro-reliefs. Therefore, Pakistan is a hub for cultivation, processes, demands, size, consumption, growth patterns, and marketing for herbs and spices at the domestic and commercial levels [83].

## 5. Conclusions

The present study revealed important information about economically important and commonly traded herbal spices. They are not only important for food, but have high therapeutic potential as well. Macroscopic and microscopic characterization will be helpful in the detection of artificial and natural deterioration and adulterants in these commonly traded spices. Plant parts were first identified macroscopically (morphological features), which is the oldest method applied for plant identification and is still considered effective and accurate. In ancient times, the organoleptic features were not only used to identify the genuineness but also to improve the quality of crude drugs. Therefore, the microscopic features were applied for the evaluation of the herbal spice taxonomy. This study also provides a baseline for herbalists in the food and herbal medicine industries. Based on the studied parameters, we recommend some modern techniques such as DNA barcoding, sequencing, phytochemical analysis, and microbiological markers for the detection of adulterants in economically important herbal spices.

## Figures and Tables

**Table 1:** List of commercial herbal spices used as food and medicine (taxa, family, spicy local name, common name and part used).

S. No	Taxa	Family	Spicy Local Name	Common Name	Part Used
1	*Allium sativum* L.	Amaryllidaceae	Lehson	Garlic	Bulb
2	*Amomum subulatum* Roxb.	Zingiberaceae	Illaichi dana kallan	Greater cardamom/Black cardamom	Seed
3	*Brassica nigra* (L.) K.Koch	Brassicaceae	Tukhm-e-Roy	Black mustard	Seed
4	*Capsicum annuum* L.	Solanaceae	Mirch	Chili pepper	Crushed fruit
5	*Cinnamomum tamala* (Buch.-Ham.) T.Nees & Eberm.	Lauraceae	Tezapatta	Indian bay leaf	Leaves
6	*Cinnamomum verum* J.Presl	Lauraceae	Darchini	True cinnamon tree	Bark
7	*Coriandrum sativum* L.	Apiaceae	Kashneez	Coriander	Mericarp
8	*Cuminum cyminum* L.	Apiaceae	Zeera sufaid	Cumin	Mericarp
9	*Curcuma longa* L.	Zingiberaceae	Haldi	Turmeric	Rhizome
10	*Elettaria cardamomum* (L.) Maton	Zingiberaceae	Illaichi Sabz	True cardamom	Fruit
11	*Foeniculum vulgare* Mill.	Apiaceae	Tukhm-e-Sounf	Fennel	Mericarp
12	*Illicium verum* Hook.f.	Schisandraceae	Badyan ka phool	Star anise	Dry fruits
13	*Mentha spicata* L.	Lamiaceae	Podina	Spearmint	Leaves
14	*Myristica fragrans* Houtt.	Myristicaceae	Jaiful	Nutmeg	Seed
15	*Myristica fragrans* Houtt.	Myristicaceae	Javitri	Mace	Fruit peel
16	*Phyllanthus emblica* L.	Phyllanthaceae	Amla khushk	Emblic	Mesocarp
17	*Piper nigrum* L.	Piperaceae	Filfil Siah	Black pepper	Fruit
18	*Punica granatum* L.	Lythraceae	Anar danna	Pomegranate seed	Seed
19	*Syzygium aromaticum* (L.) Merr. & L.M.Perry	Myrtaceae	Qaranfal	Clove	Floral bud
20	*Trachyspermum ammi* (L.) Sprague	Apiaceae	Ajwain Desi	Ajwain	Mericarp
21	*Trigonella foenum-graecum* L.	Fabaceae	Mathi Dana	Fenugreek	Seed
22	*Zingiber officinale* Roscoe	Zingiberaceae	Sonth	Ginger	Rhizome

**Table 2:** Commercial herbal spices (species, generic names, synonyms and voucher).

Species	Generic Name	Synonym	Voucher No.
*A. sativum* L.	*Allium* L.	*Allium sativum* f. *vulgare* Kazakova	131647 (ISL-QAU)
*A. subulatum* Roxb.	*Amomum* Roxb.	*Cardamomum subulatum* (Roxb.) Kuntze	131635 (ISL-QAU)
*B. nigra* (L.) K.Koch	*Brassica* L.	*Brassica nigra* f. *dentifera* Zapał.	131645 (ISL-QAU)
*C. annuum* L.	*Capsicum* L.	*Capsicum annuum* f. *luteum* Kuntze	131649 (ISL-QAU)
*C. tamala* (Buch.-Ham.) T.Nees & Eberm.	*Cinnamomum* Schaeff.	*Cinnamomum tamala* Baruah & S.C.Nath	131638 (ISL-QAU)
*C. verum* J.Presl	*Cinnamomum* Schaeff.	*Camphorina cinnamomum* (L.) Farw.	131634 (ISL-QAU)
*C. sativum* L.	*Coriandrum* L.	*Coriandrum sativum* Stolet.	131636 (ISL-QAU)
*C. cyminum* L.	*Cuminum* Tourn. ex L.	*Cuminia cyminum* J.F.Gmel.	131630 (ISL-QAU)
*C. longa* L.	*Curcuma* L.	*Curcuma longa* J.K.George & Varapr.	131639 (ISL-QAU)
*E. cardamomum* (L.) Maton	*Elettaria* Maton	*Elettaria cardamomum* Thwaites	131651 (ISL-QAU)
*F. vulgare* Mill.	*Foeniculum* Mill.	*Foeniculum vulgare* Burnat	131637 (ISL-QAU)
*I. verum* Hook.f.	*Illicium* L.	*Illicium san-ki* Perr.	131629 (ISL-QAU)
*M. spicata* L.	*Mentha* L.	*Mentha condensata* (Briq.) Greuter & Burdet	131641 (ISL-QAU)
*Myristica fragrans* Houtt.	*Myristica* Gronov.	*Aruana silvestris* Burm.f.	131631 (ISL-QAU)
*M. fragrans* Houtt.	*Myristica* Gronov.	*Aruana silvestris* Burm.f.	131648 (ISL-QAU)
*P. emblica* L.	*Phyllanthus* L.	*Cicca emblica* (L.) Kurz	131642 (ISL-QAU)
*P. nigrum* L.	*Piper* L.	*Piper nigrum* C. DC.	131633 (ISL-QAU)
*P. granatum* L.	*Punica* L.	*Punica nana* L.	131644 (ISL-QAU)
*S. aromaticum* (L.) Merr. & L.M.Perry	*Syzygium* Gaertn	*Caryophyllus aromaticus* L.	131643 (ISL-QAU)
*T. ammi* (L.) Sprague	*Trachyspermum* Link	*Ammi copticum* L.	131640 (ISL-QAU)
*T. foenum-graecum* L.	*Trigonella* L.	*Trigonella foenum-graecum* (M.Bieb.) P.Fourn.	131646 (ISL-QAU)
*Z. officinale* Roscoe	*Zingiber* Mill.	*Zingiber officinale* F.M.Bailey	131632 (ISL-QAU)

**Table 3:** Uses of spices in herbal tea, condiments and therapeutics.

Taxa	Regional Names	Ingredients	Herbal Tea	Uses in Condiments	Therapeutic Uses Other than Food
*A. sativum* L.	Lehson, sum, thom, thum, tuma and oga	Whole bulb and their powder	Garlic tea	Ketchup, garlic chatney, garam masala and salan masala	Treat itching, pain, infection associated with otitis externa as well as otitis media, otalgia, otorrhoea, furunculosis and inhance male sexuality
*A. subulatum* Roxb.	Ilaichi bari, Ilaichi surukh and Surukh ilaichi	Whole fruit and Seed	Black cardamom tea	Biryani masala	Treat cardiac arrhythmia, hypertension, nervous weakness, palpitation, typhoid fever, measles, chickenpox, dysentery, whooping, cough and inflammation of urinary bladder
*B. nigra* (L.) K.Koch	Black mustard, brown mustard, kala rai, rai-e-sarso and kala sarso	Seed	Nil	Achar masala	Treat acidity, rheumatism, piles, vomiting, relieving water retention and causing of appetizer
*C. annuum* L.	Bell pepper, daharh, surukh mirch and sru-mirch	Dried crushed Fruit and powder	Nil	In all types of chatneys, garam masala, biryani masala and salan masala	Treat stomach associated problems, tooth pain, blood circulation, hyperlipidemia and heart problems
*C. tamala* (Buch.-Ham.) T.Nees & Eberm.	Tez pat, tej pat, tamla patra and tezpata	Whole leaves and their powder	Bay leaf tea	Garam masala and biryani masala	Treat hepatic disorder, jaundice, anaemia, liver inflammation and their enlargement, heart burn, urinary bladder irritation, flu, diarrhea, immune boost and gastro associated disorders
*C. verum* J.Presl	Cinnamon, dalchini, darchini, dalchina, khog largay and khog postica	Inner bark and their powder	Cinnamon tea	Garam masala, biryani masala and salan masala	Treat the potency and strength of vital parts, leucorrhoea, weakness, paleness, blood deficiency, backache indigestion, abdominal flatulence, piles, diarrhea, toothache, fever cough, headache and heart associated disorders
*C. sativum* L.	Dhania, dhanya, dhanrhia and kashnez	Their green leaves and whole mericarp in powder form	Coriander tea	In all types of chatneys, garam masala, biryani masala and salan masala	Treat fever, hypertension, anxiety and depression, cardiac arrhythmia, palpitation, nerves associated disorder, hysteria, weakness, acidity and gastro associated problems
*C. cyminum* L.	Zeera, zira, zeera sufaid, jira, and sufaid jira	Whole mericarp and their powder	Cumin tea	Garam masala, biryani masala and salan masala	Treat gaseous distention, nausea, vomiting, acidity, heart burn, indigestion, appetizer, constipation, irritability, flatulence, dyspepsia, heartburn, vertigo, stomachache and gastrointestinal disorders
*C. longa* L.	Haldi, halda, kurkaman, haldi zard	Dried rhizome and their powder	Nil	Garam masala and biryani masala	Treat dermatological disorder, hepatobiliary diseases, peptic ulcer, psoriasis, diabetic and atherosclerosis
*E. cardamomum* (L.) Maton	Sabaz choti illiachi, elaichi, illiachi sabaz	Whole fruit	True cardamom tea	Garam masala, and biryani masala	Treat cardiac arrhythmia, cure palpitation, hysteria weakness, tachycardia and anxiety, indigestion, acidity, heartburn, stomachache abdominal cramps, diarrhea, vomiting and toothache
*F. vulgare* Mill.	Sounf, badyan, kagah and saunf	Whole mericarp and their powder	Fennel tea	Soup masala, garam masala and biryani masala	Treat indigestion, flatulence, acidity, stomachache, hepatic disorder, renal diseases, diarrhea, vomiting, toothache and abdominal cramps
*I. verum* Hook.f.	Badyani, badyan ka phool and dagad ka phool	Dried fruits and powder	Star anise tea	Garam masala, biryani masala and salan masala	Treat respiratory disorder, inflammation of lung, cough, bronchitis, flu, indigestion, acidity, stomachache, appetizer and relieve pain
*M. spicata* L.	Pudina, podina khushk, podina and badian	Aerial parts in crushed forms	Mint green tea	Chatneys, garam masala and soup	Treat abdominal cramps, gripes, flatulence, diarrhea, vomiting, toothache, heartburn, indigestion and digestive associated disorders
*M. fragrans* Houtt.	Jaifal, jaiphal, jafal, and joz	Inner parts of seed	Nil	Garam masala, biryani masala and salan masala	Treat chronic pains, arthritis, sprains, weakness, enhance sexuality, heartburn, acidity and gastro associated disorder
*M. fragrans* Houtt.	Javtri, jawtri and lawtri chilka	Dried and crushed fruit peel	Nil	Garam masala and biryani masala	Treat neuromuscular pain, articular pain, sciatic, rheumatoid, increase thickness of prostrate fluid, arthritis, weakness, heartburn, acidity and physical debility, and functional impotence
*P. emblica* L.	Amla khushk, Amla, anola and aonola	Inner region of mesocarp in powder form	Nil	Garam masala, biryani masala and salan masala	Treat immune boost, jaundice, inflammation, diarrhea, stomachache, nerves weakness, rheumatic pain, headache, chronic catarrh, flatulence and appetizer
*P. nigrum* L.	Filfil siah, filfil siyah, gol-mirch, and kali mirch	Dried fruit and their powder	Nil	Ketchup, soup, garam masala, biryani masala and salan masala	Treat indigestion, hyperacidity, nausea, vomiting, appetite, muscular pain, gaseous distention, heartburn, flatulence, hepatic disorder, stomachache, arthritis, asthma, depression, sex drive, menstrual pain, stuffy nose, dizziness and weight loos
*P. granatum* L.	Annar dana, dana-e-anar, olung dana and annar ka bijh	Dried seeds	Pomegranate tea	Garam masala and biryani masala	Treat indigestion, pain, rheumatoid arthritis, nausea, vomiting, dysentery, diarrhea, gastrointestinal cramps, cancer and cardiac disorder
*S. aromaticum* (L.) Merr. & L.M.Perry	Qarunfil, Loung, long and mikhak	Dried form of flower bud	Clove tea	Garam masala, biryani masala and salan masala	Treat expectorant, chronic pain, sex drive, flu, dyspnea, asthma, abdominal flatulence, spermatorrhoea, diarrhea, nausea and vomiting
*T. ammi* (L.) Sprague	Ajwain, Ajwain desi, ajowain, jowan and sparkiye	Whole mericarp and their powder	Ajwain desi tea	Chatneys, garam masala, biryani masala and salan masala	Treat indigestion, diarrhea, hyperacidity, nausea stomachache, vomiting, hepatic disorder, heartburn, appetizer and vertigo
*T. foenum-graecum* L.	Meethi, methi dana and methi hulba	Dried seeds and their powder	Nil	Soup, garam masala, biryani masala and salan masala	Treat indigestion, nerves and muscular pains, respiratory disorder and fever
*Z. officinale* Roscoe	Adrak, sonth, zangible and adrak sanrth	Dried rhizome and their powder	Ginger tea	Ketchup, soup, garam masala, biryani masala and salan masala	Treat indigestion, hepatic disorder, flatulence, constipation, irritability, leucorrhoea, heartburn, dyspepsia, hyperacidity, vomiting, motion sickness, diarrhea and weight loss

## Data Availability

The raw data contains the names of all participants and cannot be shared in this form.
